# Recent advances in pulmonary tuberculosis, the application of deep learning to medical topics, and highlights from this issue of *Ewha Medical Journal*

**DOI:** 10.12771/emj.2025.00395

**Published:** 2025-04-21

**Authors:** Hae-Sun Chung

**Affiliations:** Department of Laboratory Medicine, Ewha Womans University College of Medicine, Seoul, Korea

As the spring semester of 2025 begins, Korea finds itself navigating an unprecedented political crisis following the impeachment and removal of the President. Amid this national upheaval, the conflict between the government and the medical association remains unresolved. Although most medical students have formally registered for classes, academic activities have not fully returned to normal across many institutions. Despite this uncertain environment, *Ewha Medical Journal* remains committed to publishing high-quality, clinically and educationally significant research that advances medical knowledge.

Despite these challenges, we are proud to present the April 2025 issue, featuring 21 carefully selected articles: 6 review articles, 7 original research papers, and a diverse collection of other submissions. A special highlight of this issue is a series of 4 narrative review articles on recent advances in pulmonary tuberculosis (TB), offering updated insights into this crucial and evolving area of infectious disease research. Additionally, 5 original articles focus on the application of deep learning in various medical contexts, reflecting the increasing impact of artificial intelligence on healthcare. We are also pleased to include an article reporting results from a trauma survey among North Korean defectors, which we anticipate will garner international interest due to its relevance to human rights and public health. Furthermore, this issue features a health statistics paper from Statistics Korea analyzing dementia-related death statistics—a resource expected to be widely referenced by researchers, clinicians, and policymakers.

## Special topics on pulmonary tuberculosis

This April issue features a special topic section on pulmonary TB, comprising 4 comprehensive narrative reviews addressing both TB and nontuberculous mycobacterial diseases from multiple clinical and policy-oriented perspectives. Drs. Jinsoo Min, Bruno B. Andrade, Ju Sang Kim, and Yoolwon Jeong provide an extensive overview of recent innovations in TB treatment, highlighting precision medicine integration, novel and repurposed drug development, and cohort-based research to enhance therapeutic efficacy and inform policy [1]. They emphasize tailoring treatments to individual patient profiles and stages of disease progression. Dr. Jeong Uk Lim examines how pulmonary TB complicates lung cancer screening in TB-endemic areas, particularly affecting low-dose computed tomography interpretation [2]. His review underscores the necessity of improved diagnostic accuracy through artificial intelligence and biomarkers to minimize false positives and avoid unnecessary procedures. Dr. Joon Young Choi explores the increasing burden of TB-associated chronic obstructive pulmonary disease, detailing its distinct pathophysiology—characterized by persistent inflammation and structural lung damage—and reviewing evidence-based treatment options [3]. Finally, Dr. Chiwook Chung discusses current and emerging treatment strategies for *Mycobacterium avium* complex pulmonary disease, outlining existing regimen limitations, the role of antimicrobial susceptibility testing, and potential adjunctive therapies and surgical interventions [4]. Together, these reviews offer valuable clinical and research insights into the complex landscape of mycobacterial diseases.

## Two reviews and 2 original articles

This issue features 2 review articles addressing interdisciplinary healthcare topics. Dr. Dong-Ju Choi reviews telemedicine’s potential in heart failure care, highlighting benefits such as improved monitoring, reduced hospitalizations, and enhanced outcomes, while addressing challenges like digital literacy and healthcare system integration [5]. Drs. Min-Young Kim and Eun-Kyoung Pang examine associations between periodontitis and systemic diseases—including diabetes, cardiovascular diseases, and neurodegenerative conditions—emphasizing oral health’s role in systemic inflammation and advocating for collaborative care approaches [6].

Two original articles provide unique public health insights. Drs. So Hee Lee, Won Woong Lee, Haewoo Lee, Jin Yong Jun, and Jin-Won Noh analyze trauma and human rights violations among North Korean defectors, reporting high rates of post-traumatic stress disorder and depression and calling for trauma-informed support systems [7]. Drs. Sooji Hong, Bong-Kwang Jung, and Hyun-Jong Yang investigate immunogenic proteins in *Anisakis* larvae molting membranes that may trigger allergic responses in eosinophilic patients, suggesting a novel allergen source requiring further investigation [8].

## Deep learning: medical students leading innovation

This issue features 5 original research articles applying deep learning to diverse medical topics, including positron emission tomography-based organ segmentation, SHAP (Shapley Additive Explanations)-enhanced appendix cancer prediction, data imbalance modeling in diabetes, cardiac computed tomography segmentation, and blood glucose prediction using patient-provider interaction modeling. Notably, 4 of these studies were led and authored by undergraduate medical students—an exceptional achievement during widespread disruptions in medical education. These accomplishments were facilitated by the Ewha Green Ribbon Project, initiated under former Dean Eunhee Ha to maintain academic continuity and nurture future scholars. A core component of this project, the artificial intelligence and deep learning education track, was meticulously designed and implemented by Professor So Hyun Ahn. Her dedicated guidance, commitment, and generosity provided students with unique hands-on learning and research experiences in one of the most dynamic fields in medicine today. The publication of these student-led studies is a source of immense pride within our institution, reflecting scholarly achievement and innovation that commands recognition. These results affirm Ewha Womans University College of Medicine’s leadership in medical education and its standing as a model of academic excellence nationally and internationally.

## Health statistics: national trends in dementia-related mortality

Health statistics articles in *Ewha Medical Journal* primarily focus on presenting and analyzing quantitative data related to health, healthcare, and public health, aiming to deliver meaningful, data-driven insights into public health issues. In this issue, Mr. Seokmin Lee from Statistics Korea presents a comprehensive analysis of dementia-related deaths in Korea between 2013 and 2023 [9]. The study highlights a marked increase in both the number and crude rate of dementia-related deaths, especially among women and the elderly, with Alzheimer’s disease identified as the leading cause. Although age-standardized mortality has slightly decreased, the overall dementia burden continues to grow. As noted in the editorial introduction, this paper provides a robust empirical foundation that is expected to be widely cited by healthcare professionals, researchers, and policymakers for both planning and evaluation purposes. Its clarity and depth make it a valuable resource for addressing Korea’s dementia care challenges. We greatly value this contribution and look forward to further collaborations with Statistics Korea to ensure vital national health data are disseminated through rigorous academic channels.

## Guideline: Korean translation of principles of best practice and transparency

This issue includes the Korean translation of the Principles of Best Practice and Transparency in Scholarly Publishing (ver. 4), a guideline jointly developed by the Committee on Publication Ethics, the Directory of Open Access Journals (DOAJ), the Open Access Scholarly Publishing Association, and the World Association of Medical Editors [10]. Released on September 15, 2022, this document outlines essential principles for ethical and transparent scholarly publishing, covering topics such as editorial independence, peer review standards, conflicts of interest, and open access policies. The Korean translation, provided by the Korean Council of Science Editors and Infolumi, aims to make these international standards more accessible to Korean editors and publishers. By offering this translation, *Ewha Medical Journal* supports the broader dissemination of global best practices and contributes to enhancing ethical awareness and transparency within the Korean academic publishing community.

## Voices and perspectives

This issue marks a significant moment in 2025 with leadership transitions at Ewha Womans University. We congratulate Professor Hyang-Sook Lee on her inauguration as the President of Ewha Womans University, who envisions leading the university into an era of inclusive innovation for a sustainable future. We also welcome Professor Duk-Hee Kang as the 28th Dean of Ewha Womans University College of Medicine, who aims to strengthen global healthcare leadership through innovative research and education. We extend our sincere appreciation to Professor Eunhee Ha, who concluded her term as Dean in January 2025, for her exceptional dedication and service. Their messages are featured in this issue with deep respect.

This issue also includes timely and significant correspondence addressing the sharp decline in Korea’s medical research output in 2024 following the mass resignation of resident physicians. The authors analyze publication data across specialties and argue that the prolonged disruption has severely impacted both clinical and academic systems. Given the central role residents play in university hospitals, these findings underscore the structural vulnerability of Korea’s research ecosystem. This correspondence offers critical insights into one of the most urgent issues facing the Korean medical community today.

The final article introduced in this editorial was authored by Editor-in-Chief Sun Huh, reporting on *Ewha Medical Journal*’s current status regarding international indexing efforts. The journal passed the scientific evaluation by PubMed Central (PMC) and was successfully included in the DOAJ, but was not accepted by Scopus. These outcomes reflect the dedication of our editorial team, and we reaffirm our commitment to the journal’s continued growth.

In these turbulent times, we remain dedicated to publishing articles that uphold scientific rigor and social relevance. Building on the achievements of this April issue, *Ewha Medical Journal* will continue to identify timely and meaningful special topics that resonate with clinicians and researchers. We aim to foster broader collaborations with institutions such as Statistics Korea to transform valuable public data into publishable research. As a medical school-based journal, we will also strengthen educational initiatives that empower students to engage actively in research and scholarly writing. With the journal recently passing the scientific evaluation for PMC, we anticipate formal indexing soon. This milestone is expected to expand our global visibility and attract a more diverse range of submissions from around the world. We sincerely thank all authors, reviewers, and readers for their continued support and look forward to working together to advance academic excellence, global engagement, and meaningful contributions to the medical and scientific communities.
